# Predictors and Implications of Myocardial Injury in Intracerebral Hemorrhage

**DOI:** 10.1007/s00062-025-01498-4

**Published:** 2025-01-30

**Authors:** Felix Hess, Julian McGinnis, Enayatullah Baki, Tun Wiltgen, Arne Müller, Christian Maegerlein, Jan Kirschke, Claus Zimmer, Bernhard Hemmer, Silke Wunderlich, Mark Mühlau

**Affiliations:** 1 Department of Neurology, Klinikum rechts der Isar, School of Medicine and Health, Technical University of Munich, Ismaninger Str. 22, 81675 Munich, Germany; 2 Department of Computer Science, School of Computation, Information and Technology, Technical University of Munich, Munich, Germany; 3 Department of Internal Medicine I, Cardiology, School of Medicine and Health, Klinikum rechts der Isar, Technical University of Munich, München, Germany; 4 Department of Diagnostic und Interventional Neuroradiology, School of Medicine and Health, Klinikum rechts der Isar, Technical University of Munich, München, Germany; 5 Munich Cluster for Systems Neurology (SyNergy), Munich, Germany

**Keywords:** Stroke, Intracerebral hemorrhage, Troponin, Deep learning

## Abstract

**Purpose:**

Myocardial injury, indicated by an elevation of high-sensitive cardiac Troponin (hs-cTnT), is a frequent stroke-related complication. Most studies investigated patients with ischemic stroke, but only little is known about its occurrence in patients with intracerebral hemorrhage (ICH). This study aimed to assess the frequency, predictors, and implications of myocardial injury in ICH patients.

**Methods:**

Our retrospective analysis included 322 ICH patients. We defined myocardial injury as an elevation of hs-cTnT above the 99th percentile (i.e. 14 ng/L). Acute myocardial injury was defined as either a changing pattern of > 50% within 24 h or an excessive elevation of initial hs-cTnT (> 52 ng/L). 3D brain scans were assessed for ICH visually and quantitatively by a deep learning algorithm. Multiple regression models and Voxel-based Lesion-Symptom Mapping (VLSM) were applied.

**Results:**

63.0% (203/322) of patients presented with myocardial injury, which was associated with more severe strokes and worse outcomes during the in-hospital phase (*P* < 0.01). Acute myocardial injury occurred in 24.5% (79/322) of patients. The only imaging finding associated with acute myocardial injury was midline shift (69.8% vs. 44.6% for normal or stable hs-cTnT, *P* < 0.01), which also independently predicted it (odds ratio 3.29, confidence interval 1.38–7.87, *P* < 0.01). In contrast, VLSM did not identify any specific brain region significantly associated with acute myocardial injury. Acute myocardial injury did not correlate with preexisting cardiac diseases; however, the frequency of adverse cardiac events was higher in the acute myocardial injury group (11.4% vs. 4.1% in patients with normal and/or stable patterns of hs-cTnT, *P* < 0.05).

**Conclusion:**

Myocardial injury occurs frequently in ICH and is linked to poor outcomes. Acute myocardial injury primarily correlates to space-occupying effects of ICH but is less dependent on premorbid cardiac status. Nonetheless, it is associated with a higher rate of adverse cardiac events.

**Supplementary Information:**

The online version of this article (10.1007/s00062-025-01498-4) contains supplementary material, which is available to authorized users.

## Introduction

High-sensitive troponin (hs-cTn) assays are the preferred biomarker to evaluate patients with suspected acute coronary syndrome (ACS) [1]. A hs-cTn elevation indicates myocardial injury and occurs in up to 60% of all ischemic stroke patients. However, only a minority of these patients present with ACS [2–5]. Of note, a hs-cTn elevation may alternatively result from stroke-related myocardial injury. It represents the most frequent manifestation of 2018 introduced [6] and by now well-defined stroke-heart syndrome, which summarizes different, partially overlapping phenotypes of cardiac complications, especially within the first days after stroke [3, 6, 7].

In recent studies, lesion sites associated with myocardial injury were identified, particularly regions of the central autonomous network [8–10]. Furthermore, higher hs-cTn levels are associated with stroke severity, mortality, and an increased risk of future cardiovascular events [3, 7, 11, 12]. Especially acute myocardial injury might indicate a high-risk constellation of severe adverse cardiac events [5, 13]; however, precise diagnostic algorithms and therapeutic implications are still to be defined [3, 5, 14].

Although stroke-heart syndrome has attracted much attention, almost all of the available studies focused on ischemic stroke, but only a few of them investigated stroke-related myocardial injury in patients with intracerebral hemorrhage (ICH), accounting for 10–15% of all strokes [13, 15–23]. Due to its distinct pathophysiology with space-occupying effects caused by accumulation of blood and compression of surrounding brain tissue, expanding edema, as well as intraventricular extension, leading to a subsequent increase of intracranial pressure, the ICH lesion pattern associated with stroke-related myocardial injury may differ from that of ischemic stroke [15, 24, 25]. Recent studies estimate the prevalence of hs-cTn elevation between 10 and 45% in ICH patients [17, 18, 20, 22, 23]. The majority of these studies found associations of myocardial injury with poor outcomes and higher mortality [16, 20, 22, 23]. However, evidence has remained inconclusive regarding the association of myocardial injury with ICH-specific, stroke-related factors, such as stroke severity, lesion volume, lesion site, and intraventricular extension [17, 18, 20, 22]. Furthermore, a differentiation between acute and chronic myocardial injury has only been made in two studies, based on a changing pattern of hs-cTn with a reported prevalence between 9 and 29%, being associated with a worse functional outcome [20, 23].

This study sought to investigate lesion patterns of ICH associated with myocardial injury in a larger cohort of ICH patients, including a dedicated analysis of lesion locations across the entire brain on a voxel-based level. We further aimed to assess the frequency and predictors of an ICH-related acute myocardial injury and its prognostic impact on functional outcomes as well as the occurrence of adverse cardiac events.

## Material and Methods

### Patient Selection

**Fig. 1:**
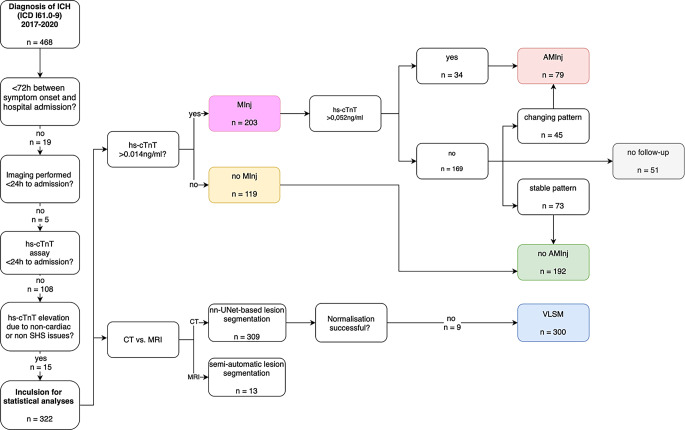
Patient selection for statistical and VLSM analyses. *n* sample size, *hs-cTnT* high-sensitive cardiac Troponin T, *SHS* stroke heart syndrome, *CT* computed tomography, *MRI* magnetic resonance imaging, *AMInj* acute myocardial injury, *MInj* myocardial injury, *VLSM* Voxel-based Lesion-Symptom Mapping

All patients admitted to our university hospital diagnosed with spontaneous ICH (I61.0–61.9; International Classification of Diseases, Tenth Revision) from January 2017 to December 2020 were retrospectively screened for the following inclusion criteria: (1) admission < 72 h after symptom onset, (2) high-sensitive cardiac Troponin T (hs-cTnT) assessment, and (3) computed tomography (CT) or magnetic resonance imaging (MRI) performed within 24 h after admission; Fig. 1 shows a flow-chart of the selection process.

### Clinical and Laboratory Variables

We collected detailed information on demographic variables, cardiovascular risk factors, premorbid cardiac status, abnormal electrocardiographic findings (ECG), and medication, such as anticoagulants and antiplatelet agents. The National Institutes of Health Stroke Scale (NIHSS) at the time of admission was used to assess stroke severity and the modified Rankin Scale (mRS) at discharge as a functional outcome measure. Laboratory parameters included platelet count, coagulation parameters, as well as renal function in terms of estimated glomerular filtration rate (eGFR, Modification of Diet in Renal Disease Equation).

A high-sensitive assay (Roche Elecsys Troponin T high sensitive) was performed for hs-cTnT measurement with an upper reference limit of the 99th percentile of a healthy reference population (14 ng/L) [1, 26]. To delineate acute myocardial injury consistent with current guidelines, we considered two scenarios likely indicating acute myocardial injury. The first scenario involves observing a changing pattern in levels of hs-cTnT. In line with international consensus statements that recommend a dynamic change between 20% and 50%, we selected a cut-off of 50% rise or fall within 24 h due to its higher specificity, particularly because our study population does not consist of patients with chest pain [27].

Furthermore, according to the current guidelines of the European Society of Cardiology, very high initial values of hs-cTnT pose a high risk for non-ST-elevation myocardial infarction (NSTEMI), being indicative of a probable acute myocardial injury. The therein-listed assay-specific cut-off value (Roche Elecsys Troponin T high sensitive 52 ng/L) was used for our study. Consequently, excessively elevated initial hs-cTnT values and/or changing patterns are referred to as acute myocardial injury [26]. Additionally, we calculated a model using acute myocardial injury defined by a 20% changing pattern to allow for better comparability with other studies [23]. The results of this analysis are presented as supplementary material (supplementary table 4).

Patients with troponin elevation due to impaired renal function (eGFR < 30 mL/min) or other cardiac diseases outside the phenotype of the stroke-heart syndrome (e.g., endocarditis) [3, 6] were excluded from statistical analysis. Adverse cardiac events were subclassified as ST-elevation ACS, ventricular dysfunction, acute rhythm disorder, and sudden cardiac death.

### Imaging

In-house CT scans were performed on either a 128-row multidetector-CT (CT Ingenuity Core 128, Philips Healthcare) or a 64-row multidetector-CT (Siemens Somatom AS+, Siemens Healthineers), MRI scans (*n* = 13) on a 3 T Scanner (Achieva dStream Philips) following standard stroke protocols.

In addition to employing deep-learning algorithms to determine lesion volume and conducting Voxel-based Lesion-Symptom Mapping (VLSM) analyses (explained later), we screened neuroradiological reports for the following easily assessable imaging features related to ICH, indicating a space-occupying effect, and/or suggesting broader impacts beyond the lesion’s location: affected hemisphere (right, left, infratentorial), deep brain ICH (compared to lobar), perihematomal edema, intraventricular extension, acute hydrocephalus, and midline shift.

#### Lesion Segmentation

**Fig. 2:**
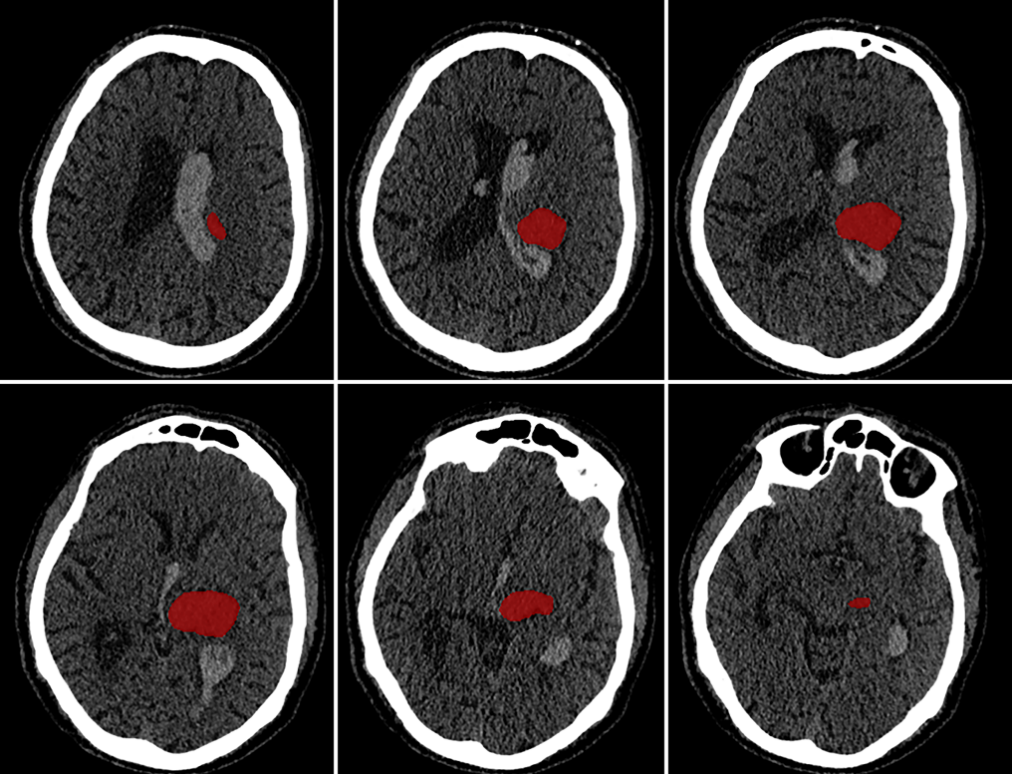
CT scan of a patient with ICH and intraventricular extension. Lesion segmentation of intraparenchymal blood performed using the nnU-Net 3D full-resolution network trained in the context of this study

For pre-segmentation of intraparenchymal lesions (i.e. ICH) on CT Scans, a nnU-Net 3D full-resolution network (Isensee et al. 2021) was trained and employed ([28, 29]; Fig. 2, Supplementary Methods, Supplementary Table 1). The code has been made available on github.com (https://github.com/jqmcginnis/ich_segmentation). Lesion masks were visually controlled and manually corrected, if necessary, using ITK-SNAP (Version 4.0.2, www.itksnap.org). On MRI scans, ITK-SNAP was applied for manual delineation of lesions using FLAIR images (repetition time = 4800 ms, echo time = 282 ms, inversion time = 1650 ms, field of view 250 × 250 × 200 mm, 1 mm^3^ voxels). Lesion volumes were calculated using FSL (FMRIB Software Library, Version 6.0.6).

### Voxel-based Lesion-Symptom Mapping

We intended to include all patients with available CT scans for VLSM (*n* = 309). Nine patients were excluded from VLSM analyses due to unsuccessful normalization related to either issues with imaging quality (*n* = 6) or the presence of solely intraventricular hemorrhage without accompanying intraparenchymal ICH (*n* = 3). The scans, as well as respective lesion masks, were warped into 1 × 1 × 1 mm stereotaxic space using the CT-normalize function, available in the Clinical Toolbox of the Statistical Parametric Mapping package (version 12; SPM12; Wellcome Trust Centre for Neuroimaging, University College London, London, UK; http://www.fil.ion.ucl.ac.uk/spm), implemented in MATLAB (Version R2022a. Natick, Massachusetts: The MathWorks Inc). VLSM analyses were conducted on the NiiStat toolbox (RRID:SCR_014152, www.nitrc.org/projects/niistat), requiring MATLAB and SPM. Only voxels lesioned in at least ten patients were included for statistical analysis [30, 31]. Liebermeister test with 4000 permutations was performed for non-parametric mapping, using a false discovery rate (FDR) of 5% (*P* < 0.05) to correct for multiple comparisons [30, 32]. To improve the anatomical validity, a correction for lesion volume was conducted [33]. We calculated two different VLSM models, both with hs-cTnT as a binary variable of interest: 1) Myocardial injury (hs-cTnT > 14 ng/L) vs. no myocardial injury (i.e., normal values of hs-cTnT); 2) Acute myocardial injury (i.e. very high initial values and/or a changing pattern of hs-cTnT) vs. no acute myocardial injury (normal values of hs-cTnT or moderately elevated values, i.e. 15–52 ng/L with a stable pattern; Fig. 1). To evaluate statistical power, we created lesion frequency maps for both of our VLSM models, running the Python-based Matplotlib library (version 3.6.2). Subsequently, lesion overlays and statistical maps were warped to a standard template (Montreal Neurological Institute 152 standard space template, voxel size of 1 × 1 × 1 mm^3^), using the greedy tool [34].

### Statistical Analysis

Statistical analyses were performed using IBM SPSS Statistics (Version 29.0.1). Datasets were categorized following the same approach as for VLSM analyses, comparing (1) Myocardial injury with no myocardial injury and (2) acute myocardial injury with no acute myocardial injury (i.e., normal values of hs-cTnT or moderately elevated values with a stable pattern). A two-sample t‑test was used for analyses of metric data, the Mann-Whitney‑U Test for ordinal data, and the chi-square test for binary categorical variables. Furthermore, a binomial logistic regression analysis was calculated to investigate the predictive value of parameters available from anamnesis and diagnostic workup in the acute stroke setting. We aimed to include variables that were significantly associated with acute myocardial injury in our single-variable analyses. Based on existent evidence of associations with myocardial injury, we also incorporated lesion volume [20, 23], intraventricular extension [18], and lesion location (deep vs. lobar ICH) [22]. The threshold for statistical significance was defined as a two-sided *p*-value of < 0.05.

## Results

A total of 322 patients (52.8% male) with a mean age of 72.0 years (± 13.9) were included for statistical analyses (Fig. 1). Myocardial Injury occurred in 63.0% (203/322) of all patients and 24.5% (79/322) were classified as acute myocardial injury. The overall frequency of adverse cardiac events was 5.6% (18/322). Among these, acute rhythm disorders were the most common (*n* = 10, particularly newly diagnosed atrial fibrillation after stroke, AFAS *n* = 7), whereas ST-elevation ACS (*n* = 2), acute ventricular dysfunction (*n* = 3), and sudden cardiac death (*n* = 3) were less frequent. Most adverse cardiac events (72.2%) occurred within the first 48 h after symptom onset. The remainder, still the remarkable proportion of almost one-third, occurred significantly later (day 4–13) primarily comprising treatment-requiring arrhythmias (ventricular tachycardia, two instances of treatment-requiring hemodynamically unstable tachyarrhythmia absoluta, and one instance of bradyarrhythmia absoluta in the context of atrial fibrillation).

### Demographic, Clinical, and Radiological Parameters

The results of univariate analyses on patients with myocardial injury vs. no myocardial injury are presented in Table 1 and 2 compares acute myocardial injury vs. no acute myocardial injury (i.e. normal values or elevated but stable patterns of hs-cTnT). Both, patients with myocardial injury (Table 1) and acute myocardial injury (Table 2) exhibited more severe strokes, as indicated by higher scores on the NIHSS.

**Table 1:** Overview of demographic, clinical, and imaging variables. Study cohort dichotomized to hs-cTnT-levels (99th percentile)

	No myocardial injury (hs-cTnT ≤ 14 ng/L) (*n* = 119)	Myocardial injury (hs-cTnT > 14 ng/L) (*n* = 203)	Nominal *p*-value
Pre-existing conditions			
Age, y mean (SD)	69.0 (± 13.9)	73.8 (± 13.7)	*<* *0.01*
Sex, male (%)	47.1	56.2	0.11
Premorbid mRS, median (IQR)	1 (0–1)	1 (1–2)	*<* *0.01*
Hypertension (%)	82.4	85.7	0.42
Hypercholesterolemia (%)	28.0	31.3	0.60
Diabetes (%)	11.8	19.9	0.06
Previous stroke (%)	19.3	22.7	0.48
Antiplatelet drugs (%)	25.2	30.1	0.35
Oral anticoagulants (%)	16.0	29.1	*<* *0.01*
Coronary heart disease (%)	12.6	17.2	0.185
Atrial fibrillation (%)	15.1	31.5	*<* *0.01*
Structural cardiac disease (%)	10.1	15.8	0.15
Baseline clinical and radiological parameters			
NIHSS, median (IQR)	13 (6–20)	18 (10–29)	*<* *0.01*
ECG changes (%)	32.1	53.6	*<* *0.01*
ICH Volume, mL mean (SD)	42.2 (± 43.8)	47.4 (± 47.2)	0.30
Hemisphere, right (%)	49.5	50.0	0.94
Deep Brain ICH (vs. lobar, %)	42.4	49.8	0.20
Perihematomal edema (%)	88.0	86.1	0.66
Intraventricular extension (%)	47.1	57.1	0.08
Acute hydrocephalus (%)	26.3	32.5	0.24
Midline shift (%)	46.4	64.2	*<* *0.01*
Outcome parameters			
mRS at discharge, median (IQR)	5 (3–6)	5 (4–6)	*<* *0.01*
Mortality (%)	27.7	40.1	0.03
Adverse cardiac events (%)	0.8	8.4	*<* *0.01*

*SD* standard deviation, *IQR* interquartile range, *n* sample size, *ICH* intracerebral hemorrhage, *hs-cTnT* high-sensitive cardiac troponin T, *y* years, *NIHS*S National Institute of Health Stroke Severity Scale, *mRS* modified Rankin Scale, *ECG* electrocardiogram, *mL* milliliters, *CI* confidence interval

**Table 2:** Demographic, clinical, and imaging findings in patients with acute myocardial injury compared to patients with normal or moderately elevated values of hs-cTnT and stable patterns

	No acute myocardial injury (*n* = 192)	Acute myocardial injury (*n* = 79)	Nominal *p*-value
Pre-existing conditions			
Age, y mean (SD)	70.2 (± 13.7)	73.7 (± 14.7)	0.14
Sex, male (%)	52.3	53.2	0.90
Premorbid mRS, median (IQR)	1 (0–1)	1 (0–2)	*0.02*
Hypertension (%)	82.6	86.1	0.48
Hypercholesterolemia (%)	32.5	31.7	0.89
Diabetes (%)	16.5	24.1	0.16
Previous stroke (%)	19.2	19.0	0.97
Antiplatelet drugs (%)	27.3	24.0	0.58
Oral anticoagulants (%)	22.1	22.8	0.82
Coronary heart disease (%)	16.9	10.1	0.20
Atrial fibrillation (%)	20.4	30.4	0.08
Structural cardiac disease (%)	14.5	15.2	0.89
Baseline clinical and radiological parameters			
NIHSS, median (IQR)	13 (5–20)	18 (9–22)	*0.03*
ECG changes (%)	41.4	54.3	0.10
ICH Volume, mL mean (SD)	36.2 (± 39.1)	42.7 (± 43.2)	0.52
Hemisphere, right (%)	50.4	44.6	0.44
Deep brain ICH (vs. lobar %)	46.8	51.9	0.45
Perihematomal edema (%)	85.8	87.2	0.77
Intraventricular extension (%)	48.3	59.5	0.10
Acute hydrocephalus (%)	26.9	24.1	0.63
Midline shift (%)	44.6	69.8	*<* *0.01*
Outcome parameters			
mRS at discharge, median (IQR)	4 (3–5)	5 (4–6)	*<* *0.01*
Mortality (%)	24.4	36.7	*0.04*
Adverse cardiac events (%)	4.1	11.4	*0.03*

*SD* standard deviation, *IQR* interquartile range, *n* sample size, *ICH* intracerebral hemorrhage, *AMInj* acute myocardial injury, *y* years, *NIHS*S National Institute of Health Stroke Severity Scale, *mRS* modified Rankin Scale, *ECG* electrocardiogram, *mL* milliliters, *CI* confidence interval

Myocardial injury appeared to be more dependent on pre-existing conditions (e.g., premorbid status, medication use) compared to acute myocardial injury. Midline shift was the only imaging finding significantly associated with myocardial injury and acute myocardial injury, and emerged as the primary independent predictor of acute myocardial injury, as evidenced by the binomial logistic regression analysis (Table 3), which was calculated to assess the predictive value for acute myocardial injury of parameters available from standard diagnostic workup within the first hours after hospital admission, that have been associated with acute myocardial injury in our single-variable analyses (i.e. premorbid mRS, NIHSS on admission and midline shift) and/or in previous studies (models *p*-value = 0.024). Patients with myocardial injury, including those with acute myocardial injury, experienced worse functional outcomes and higher mortality during the in-hospital phase, as well as a greater rate of adverse cardiac events (Tables 1 and 2). In contrast, a changing pattern of hs-cTnT levels with a 20% cut-off did not effectively identify a subgroup of patients with poorer outcomes and increased mortality in our cohort (Supplementary Table 4).

**Table 3:** Multivariate binomial logistic regression analysis for the occurrence of an acute myocardial injury including parameters available from anamnestic and acute diagnostic workup

Independent parameter	*β*-coefficient	Odds Ratio	95% CI	*p*-value
Premorbid mRS	0.27	1.31	0.99–1.73	0.940
NIHSS	−0.01	0.99	0.95–1.03	0.67
Deep brain ICH (vs. lobar)	−0.07	0.812	0.44–1.96	0.85
Intraventricular extension	0.19	1.21	0.59–2.45	0.60
Midline Shift	1.19	3.29	1.38–7.87	*<* *0.01*
ICH Volume	0.00	0.99	0.98–1.01	0.053

*NIHSS* National Institute of Health Stroke Severity Scale, *mRS* modified Rankin Scale, *CI* confidence interval

### Results of the VLSM Models

300 patients were included for VLSM (Fig. 1). Lesion distribution was nearly symmetrically across the whole brain (42% left vs. 40% right hemispheric, 18% infratentorial hemorrhages). Lesion frequency maps of the VLSM analyses are demonstrated in Fig. 2. Neither the first VLSM analysis (*n* = 300), investigating myocardial injury as a binary variable (vs. normal values of hs-cTnT) nor the second VLSM analysis (*n* = 232), investigating acute myocardial injury as a binary variable (vs. normal or moderately elevated values of hs-cTnT with stable pattern), revealed any significant voxels (FDR-corrected *P* < 0.05). A correction for lesion volume in two additional VLSM analyses did not change these results (Supplementary Tables 2 and 3).

## Discussion

This study examined the frequency and predictors of myocardial injury and its effects on clinical outcomes in a large cohort of ICH patients. A deep learning-based framework for lesion segmentation was used to reliably determine lesion volumes and VLSM was performed to identify specific lesion sites associated with myocardial injury. Further, we especially attempted to distinguish between pre-existing, chronic, and acute myocardial injury to identify ICH-driven mechanisms of acute myocardial injury and its complications.

Myocardial injury is a frequent finding in ICH. With an all-over frequency of 63% in our cohort, it occurred even more often than in previous studies on ICH [13, 16–18, 20, 22], and the majority of analyses on stroke-heart syndrome in ischemic stroke [2, 6, 7, 9]. It is also worth noting that our research found acute myocardial injury in 24.5% of our patients, i.e. almost in every fourth patient. Consistent with the literature, older age, and poorer premorbid health status were observed in ICH patients with myocardial injury [17], whereas acute myocardial injury was less dependent on pre-existing conditions (Tables 1 and 2). Also in line with prior studies, patients with myocardial injury had higher scores on the NIHSS and a worse prognosis in terms of mortality and functional outcome [16, 18, 20, 22, 23], which applied likewise to the acute myocardial injury cohort. The evidence of pre-existing cardiac diseases impacting an ICH-related myocardial injury appears controversial in the literature [13, 20, 22, 23]. Even though patients with cardiac diseases and ECG changes had higher troponin levels in our cohort (Table 1), the development of acute myocardial injury was statistically not dependent on the premorbid cardiac status (Table 2), pointing toward a stronger causal relationship with the ICH itself [6, 35]. While acute myocardial injury may primarily result from ICH in many cases, clinicians should remain vigilant for additional cardiac pathologies that may require intervention, since patients with acute myocardial injury still showed a significantly higher rate of adverse cardiac events (11% vs. 4%).

**Fig. 3:**
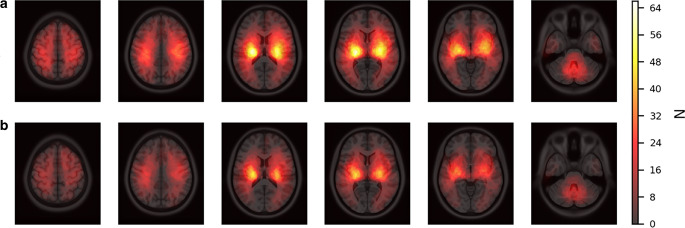
Lesion frequency maps of all patients included in VLSM analyses. **a** Lesion overlay of the first VLSM analysis (*n* = 300) with MInj as a binary variable, **b** Lesion overlay of the second VLSM analysis (*n* = 232) comparing patients with AMInj to those with normal or moderately elevated values of hs-cTnT with a stable pattern

Especially for ischemic stroke, the understanding of lesion location impacting cardiac dysfunction and troponin elevation is continuously improving [3]. Stroke lesions affecting parts of the central autonomous network, primarily the insular cortex, can cause autonomic dysfunctions leading to various cardiac complications [9, 10, 36, 37]. Regarding ICH, the data remain controversial. Qin et al. found an association of the right insular cortex as well as the thalamus with ECG changes and an elevation of cardiac markers (creatine kinase and CK-MB), but not with an elevation of cardiac troponin, the preferred biomarker for myocardial injury [1, 17]. In other cohorts, myocardial injury was more frequent in deep brain ICH patients and left hemispheric ICH [18, 22], which could not be reproduced in our cohort (Table 1). To the best of our knowledge, this is the first study that investigated the impact of lesion location on ICH-related myocardial injury on a voxel-based level. Both of our VLSM analyses did not detect any voxels significantly associated with myocardial injury as well as acute myocardial injury, although there was a sufficient lesion overlap [31, 33] in all brain regions ever associated with a troponin increase in any subtype of stroke, suggesting sufficient statistical power (Fig. 3; [3, 17, 18, 22]). VLSM, based on CT and MRI data, is a powerful, often-validated method for stroke imaging, including studies on ICH [31, 33, 38, 39]. In cohorts as large as ours, it is known to be more likely to identify even small and irrelevant effects by VLSM than to miss meaningful results [30, 40]. Furthermore, a correction for lesion volume, which was also performed in our study, improves the anatomical validity, providing even more reliable results [17, 18, 22, 33]. Finally, the rather liberal configuration of statistical testing with 4000 permutations and FDR instead of Bonferroni as correction for multiple comparisons should be mentioned [32]. In summary, although the interpretation of negative findings requires caution, our results suggest that lesion location as a single variable may not be as predictive for the development of myocardial injury in ICH as in ischemic stroke.

Myocardial injury, especially acute myocardial injury, was more frequent in patients with midline shift on imaging, remaining the main independent predictor of acute myocardial injury in our multiple regression analysis with parameters of the acute diagnostic workup (Table 3). Even though it is an easily assessable parameter indicating space-occupying processes, only a few studies evaluated its association with myocardial injury, yielding results compatible with ours [18]. We believe that the space-occupying component of intracerebral hemorrhage (ICH), with local compression of surrounding tissue and increased intracranial pressure, represents a decisive and common mechanism for disturbing the hypothalamic-pituitary-adrenal axis and other components of the central autonomic network, leading to autonomic dysfunction and thereby causing acute myocardial injury [25, 35, 37]. Since other imaging parameters linked to increased intracranial pressure, such as lesion volume, perihematomal edema, or intraventricular extension, exhibited no correlation with myocardial injury, we assume that a composite of these factors delineates the magnitude of the hemorrhage’s space-occupying impact, best expressed by midline shift as a sole variable. This interpretation is also compatible with the negative results of our VLSM analyses.

We acknowledge the limitations of our study. In addition to its retrospective and single-center design, our cohort’s rate of critically ill patients was comparatively high, which may lead to an overestimation of the frequency of myocardial injury and acute myocardial injury. Additionally, in 25% of all cases, hs-cTnT values were missing, possibly introducing some bias. Furthermore, our main finding regarding the strong predictive value of midline shift requires replication in an independent cohort.

We conclude that patients with severe, space-occupying ICH are at significantly increased risk of developing acute myocardial injury, which, as a hemorrhage-heart syndrome, is an immediate result of the ICH and less dependent on premorbid cardiac status. These patients still have a higher risk of cardiac complications and, overall, a poorer prognosis. As a possible consequence, cardiac troponin may become part of a standardized follow-up in the acute phase of ICH, e.g. 24 h after admission. Given the higher frequency of adverse cardiac events (11% in our cohort) and their time course, identified patients may require more thorough and extended monitoring of cardiovascular parameters and may benefit from a cardiological workup.

## Supplementary Information

The supplementary material contains detailed information about the training data of our nnU-Net used for lesion segmentation, as well as the results of VLSM. Furthermore, it includes a table presenting the results of an additional analysis using a 20% changing pattern to define acute myocardial injury, allowing for better comparability with other studies.

## Data Availability

The data that support the findings of this study are available on reasonable request from the corresponding author.
