# Supplementary material for: Predictors and Implications of Myocardial Injury in Intracerebral Hemorrhage
Source: Clin Neuroradiol. 2025 Jan 30;35(2):395–404. doi: 10.1007/s00062-025-01498-4 (PMC12174305; doi:10.1007/s00062-025-01498-4)
Supplement: Supplementary file 1 — The supplementary material contains detailed information about the training data of our nnU-Net used for lesion segmentation, as well as the results of VLSM. Furthermore, it includes a table presenting the results of an additional analysis using a 20% changing pattern to define acute myocardial injury, allowing for better comparability with other studies. [file 62_2025_1498_MOESM1_ESM.docx]

**Supplementary Methods**

Training data of nnU-Net-based models for lesion segmentation

For volumetric analysis of intracerebral hemorrhage (ICH), we manually segmented CT scans of N=100 patients with a representative distribution of ICH severity using ITK-SNAP (Yushkevich, PA et al., 2006), and split the dataset according to common machine learning protocols into training set n_train=80, and test set n_test=20.

To obtain an automated segmentation pipeline, we used the established nnU-Net V2 framework (Isensee F et al., 2021) and trained two different segmentation networks, i.e. a 2D nnU-Net and a full resolution 3D-nnU-Net, with five-fold cross-validation using only the predefined training set. To assess the quality of segmentation results, we used the hold out test set (N=20) and compared the predictions of the 2D and 3D full resolution networks against the manually segmented ground truth using anima toolkit, specifically the animaSegPerfAnalyzer (Commowick, O et al., 2018).

Following the comparative analysis, the nnU-Net 3D full-resolution network was identified as the superior method (Supplementary Table 1) and consequently applied to segment the scans within our total cohort.

**Supplementary Table 1.** Comparison of networks trained for automated lesion segmentation

| Metric | nnU-Net 2D Mean ± SD | nnU-Net 3D Mean ± SD |
| --- | --- | --- |
| Jaccard | 0.66 ± 0.28 | 0.73 ± 0.25 |
| Dice | 0.75 ± 0.29 | 0.81 ± 0.24 |
| Sensitivity | 0.71 ± 0.26 | 0.77 ± 0.26 |
| Specificity | 0.99 ± 0.00 | 0.99 ± 0.00 |
| Positive predictive value | 0.85 ± 0.28 | 0.88 ± 0.21 |
| Negative predictive value | 0.99 ± 0.00 | 0.99 ± 0.00 |

Abbreviations: SD = standard deviation

**Supplementary Table 2.** Test data of the first VLSM analyses comparing MInj to no MInj.

| Test data of the first VLSM analysis with (left) and without (right) correction for lesion volume | |
| --- | --- |
| *Only analyzing voxels non-zero in at least 10 individuals.*  *Analyzing results_test with 300 participants for binary variable TnT_Elevation across 716959 voxels.*  *Computing Liebermeister measures for 716959 voxels with 1 binary variable (positive Z when 0 voxels have behavior 0 and 1 voxels have behavior 1).*  *4000 permutations required 1493.1081 seconds*  *Thresholds are one-tailed (we hypothesized that injured tissue will , if at all, cause higher TnT values (but not prevent TNT increase)*  *p<0.050 permutation correction for TnT_Elevation correspond to the voxel-wise threshold of z>4.32158*  *Range of observed voxel-wise z-values:-3.598858 to 3.542912,*  *0 voxels survive threshold* | *Only analyzing voxels non-zero in at least 10 individuals.*  *Analyzing results_test with 300 participants for binary variable TnT_Elevation across 716959 voxels.*  *Computing Liebermeister measures for 716959 voxels with 1 binary variable (positive Z when 0 voxels have behavior 0 and 1 voxels have behavior 1).*  *4000 permutations required 1493.1081 seconds*  *Thresholds are one-tailed (we hypothesized that injured tissue will , if at all, cause higher TnT values (but not prevent TNT increase)*  *p<0.050 permutation correction for TnT_Elevation correspond to the voxel-wise threshold of z>4.32158*  *Range of observed voxel-wise z-values:-3.598858 to 3.542912,*  *0 voxels survive threshold* |

Abbreviations: VLSM = Voxel-based Lesion-Symptom Mapping, MInJ = Myocardial Injury,

TnT = Troponin T

**Supplementary Table 3.** Test data of the second VLSM analyses comparing AMInj to no AMInj.

| Test data of the second VLSM analysis with (left) and without (right) correction for lesion volume | |
| --- | --- |
| *Only analyzing voxels non-zero in at least 10 individuals.*  *Analyzing results_test with 232 participants for binary variable AMInj across 434172 voxels.*  *Computing Liebermeister measures for 434172 voxels with 1 binary variable (positive Z when 0 voxels have behavior 0 and 1 voxels have behavior 1).*  *4000 permutations required 736.1993 seconds*  *Thresholds are one-tailed (we hypothesized that injured tissue will , if at all, cause higher probability of AMInj_ (but not prevent AMInj)*  *p<0.050 permutation correction for AMInj correspond to the voxel-wise threshold of z>3.77477*  *Range of observed voxel-wise z-values =-3.845543 to 2.537429,*  *0 voxels survive threshold* | *Only analyzing voxels non-zero in at least 10 individuals.*  *Analyzing results_test with 232 participants for binary variable AMInj across 434172 voxels.*  *Computing Liebermeister measures for 434172 voxels with 1 binary variable (positive Z when 0 voxels have behavior 0 and 1 voxels have behavior 1).*  *4000 permutations required 736.1993 seconds*  *Thresholds are one-tailed (we hypothesized that injured tissue will , if at all, cause higher probability of AMInj_ (but not prevent AMInj)*  *p<0.050 permutation correction for AMInj correspond to the voxel-wise threshold of z>3.77477*  *Range of observed voxel-wise z-values =-3.845543 to 2.537429,*  *0 voxels survive threshold* |

Abbreviations: VLSM = Voxel-based Lesion-Symptom Mapping, AMInJ = Myocardial Injury

**Supplementary Table 4.** Demographic, clinical, and imaging findings in patients with acute myocardial injury based only on the >20% changing pattern criterion of hs-cTnT compared to patients with normal and/or stable pattern of hs-cTnT

|  | **No elevation and/or stable pattern of**  **hs-cTnT**  (n = 194) | **Changing pattern**  **of hs-cTnT**  (n = 77) | **nominal**  **p-value** |
| --- | --- | --- | --- |
| Pre-existing conditions | | | |
| Age, y mean (SD) | 71.4 (±13.9) | 71.9 (±14.0) | 0.39 |
| Sex, male (%) | 51.4 | 54.5 | 0.64 |
| Premorbid mRS, median (IQR) | 1 (0-2) | 1 (0-2) | 0.46 |
| Hypertension (%) | 82.3 | 84.4 | 0.68 |
| Hypercholesterolemia (%) | 30.0 | 33.3 | 0.25 |
| Diabetes (%) | 16.6 | 22.7 | 0.25 |
| Previous stroke (%) | 21.0 | 15.6 | 0.31 |
| Antiplatelet drugs (%) | 27.6 | 24.7 | 0.62 |
| Oral anticoagulants (%) | 21.5 | 22.1 | 0.93 |
| Coronary heart disease (%) | 16.6 | 10.4 | 0.35 |
| Atrial fibrillation (%) | 22.1 | 26.0 | 0.5 |
| Structural cardiac disease (%) | 12.7 | 18.2 | 0.25 |
| Baseline clinical and radiological parameters | | | |
| NIHSS, median (IQR) | 16.1 (7-21) | 15.3(7-20) | 0.99 |
| ECG changes (%) | 39.1 | 57.1 | **0.02** |
| ICH Volume, mL mean (SD) | 39.9 (±42.6) | 35.9 (±33.7) | 0.37 |
| Hemisphere, right (%) | 52.0 | 39.4 | 0.29 |
| Deep brain ICH (vs. lobar %) | 46.1 | 49.4 | 0.63 |
| Perihematomal edema (%) | 87.6 | 81.6 | 0.18 |
| Intraventricular extension (%) | 49.2 | 58.4 | 0.17 |
| Acute hydrocephalus (%) | 27.8 | 23.4 | 0.46 |
| Midline shift (%) | 48.9 | 63.5 | **0.05** |
| Outcome parameters | | | |
| mRS at discharge, median (IQR) | 4 (3-5) | 4 (4-6) | 0.66 |
| Mortality (%) | 28.2 | 31.2 | 0.63 |
| Adverse cardiac events (%) | 3.9 | 10.4 | **0.04** |

Abbreviations: SD = standard deviation, IQR = interquartile range, n = sample size, ICH = intracerebral hemorrhage, hs-cTnT = high-sensitive cardiac troponin T, y = years, NIHSS = National Institute of Health Stroke Severity Scale, mRS = modified Rankin Scale, ECG = electrocardiogram, mL = milliliters, CI = confidence interval
